# Primary paediatric epidural sarcomas: molecular exploration of three cases

**DOI:** 10.1186/s12885-019-5368-z

**Published:** 2019-02-28

**Authors:** Sharon Y. Y. Low, Chik Hong Kuick, Wan Yi Seow, Nurfahanah Bte Syed Sulaiman, Huiyi Chen, Derrick W. Q. Lian, Kenneth T. E. Chang, Enrica E. K. Tan, Shui Yen Soh, Grace I. L. Tan, Lee Ping Ng, Wan Tew Seow, David C. Y. Low

**Affiliations:** 1 Neurosurgical Service, KK Women’s and Children’s Hospital, Singapore, Singapore; 2 Department of Pathology and Laboratory Medicine, KK Women’s and Children’s Hospital, Singapore, Singapore; 3 Paediatric Haematology/Oncology Service, KK Women’s and Children’s Hospital, Singapore, Singapore; 4 VIVA-KKH Brain and Solid Tumours Laboratory, 100 Bukit Timah Road, Singapore, 229899 Singapore; 5 Department of Neurosurgery, National Neuroscience Institute, 11 Jalan Tan Tock Seng, Singapore, 308433 Singapore; 6 SingHealth Duke-NUS Neuroscience Academic Clinical Program, 11 Jalan Tan Tock Seng, Singapore, 308433 Singapore

**Keywords:** Epidural sarcoma, Next-generation sequencing

## Abstract

**Background:**

Primary paediatric epidural sarcomas are extremely rare. Overall, there remains a paucity of knowledge in paediatric epidural sarcomas owing to the infrequent number of cases. The Archer FusionPlex Sarcoma Kit (ArcherDX, Inc) is a next-generation sequencing assay that has been reported to be a useful technique to detect recurrent fusion in sarcomas. We report the molecular exploration of 3 primary paediatric epidural sarcomas—one in the cranium (mesenchymal chondrosarcoma) and 2 in the spine (mesenchymal chondrosarcoma and Ewing sarcoma respectively).

**Case presentation:**

This is a study approved by the hospital ethics board. Clinico-pathological information from 3 consenting patients with primary epidural sarcomas was collected. These selected tumours are interrogated via Archer FusionPlex Sarcoma Kit (ArcherDX, Inc) for genomic aberrations. Results were validated with RT-PCR and Sanger sequencing. All findings are corroborated and discussed in concordance with current literature. Our findings show 2 variants of the HEY1-NCOA2 gene fusion: HEY1 (exon 4)-NCOA2 (exon 13) and HEY1 (exon 4)-NCOA2 (exon 14), in both mesenchymal chondrosarcoma patients. Next, the Ewing sarcoma tumour is found to have EWSR1 (exon 10)-FLI1 (exon 8) translocation based on NGS. This result is not detected via conventional fluorescence in situ testing.

**Conclusions:**

This is a molecularly-centered study based on 3 unique primary paediatric epidural sarcomas. Our findings to add to the growing body of literature for these exceptionally rare and malignant neoplasms. The authors advocate global collaborative efforts and in-depth studies for targeted therapy to benefit affected children.

## Background

Primary paediatric epidural sarcomas are extremely rare and little is known about such tumours. Gene fusions are an important category of driver mutations in paediatric sarcomas [1]. While well-characterized and commonly occurring gene fusions can be identified by standard laboratory assays such as FISH and RT-PCR, an advanced technique such as next-generation sequencing (NGS) is often required to identify rare or novel gene fusions [2]. Gene fusion identification serves to confirm a pathological diagnosis, and is also important in relation to treatment as certain gene fusions have drug-targetable domains [3].

In this study, we report clinical, pathological and molecular features of three unique epidural sarcomas presenting with neurological compromise located in the cranium and spine. We describe the use of a next-generation sequencing-based assay (Archer FusionPlex Sarcoma assay (ArcherDX, Inc)) as a technique to identify gene fusions in these three primary paediatric epidural sarcomas [2, 4, 5].

## Case presentation

### Case 1: Cranial epidural mesenchymal chondrosarcoma

**Fig. 1:**
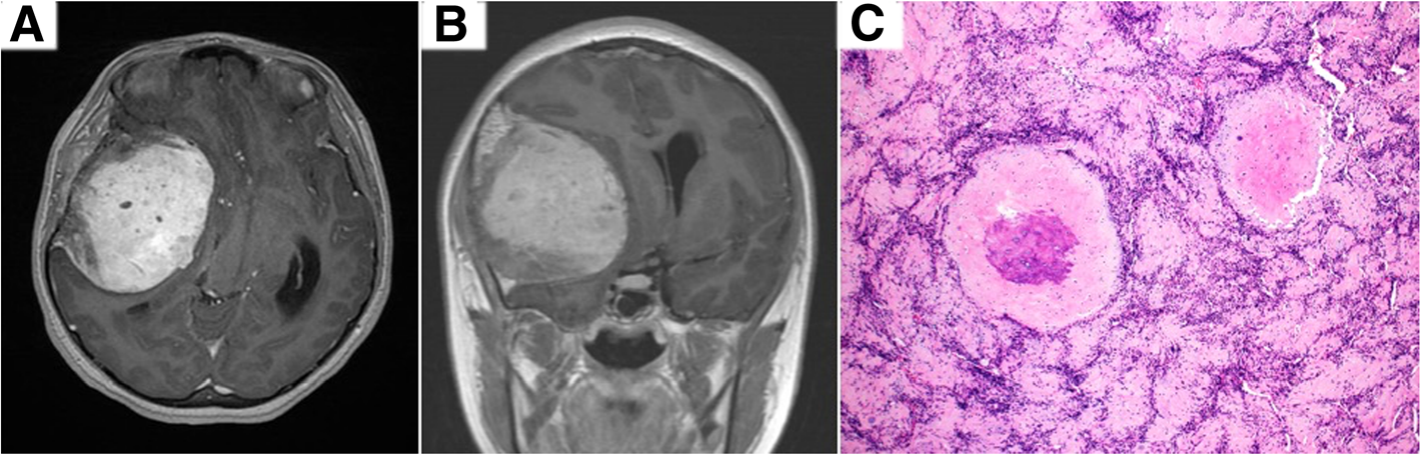
Representative post-contrast T1-weighted MRI images in axial (**a**) and coronal (**b**) demonstrating a right extra-axial, fronto-temporal lesion causing mass effect and midline shift. **c** Haematoxylin and eosin stain slide (× 100) shows tumour tissue with a bimorphic pattern, composed of primitive-looking spindle and round cells interspersed with islands of neoplastic cartilage. In many areas, the cartilage demonstrates hyalinization and secondary ossification

**Fig. 2:**
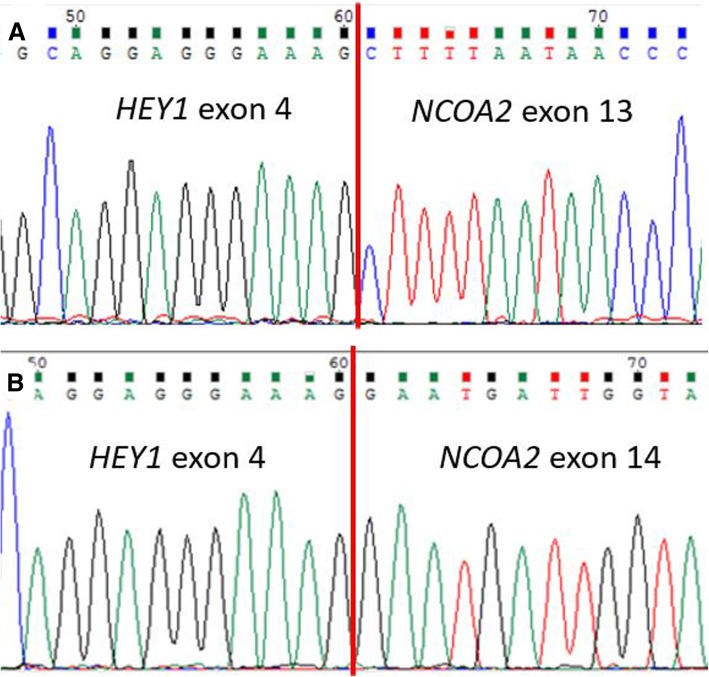
Representative post-contrast T1-weighted MRI images in sagittal (**a**) and axial (**b**) demonstrating an enhancing intradural, extramedullary lesion with dura thickening at L2. **c** Haematoxylin and eosin stain slide (× 100) depicting round to spindle cells, in association with cartilage and bone formation

**Fig. 3:**
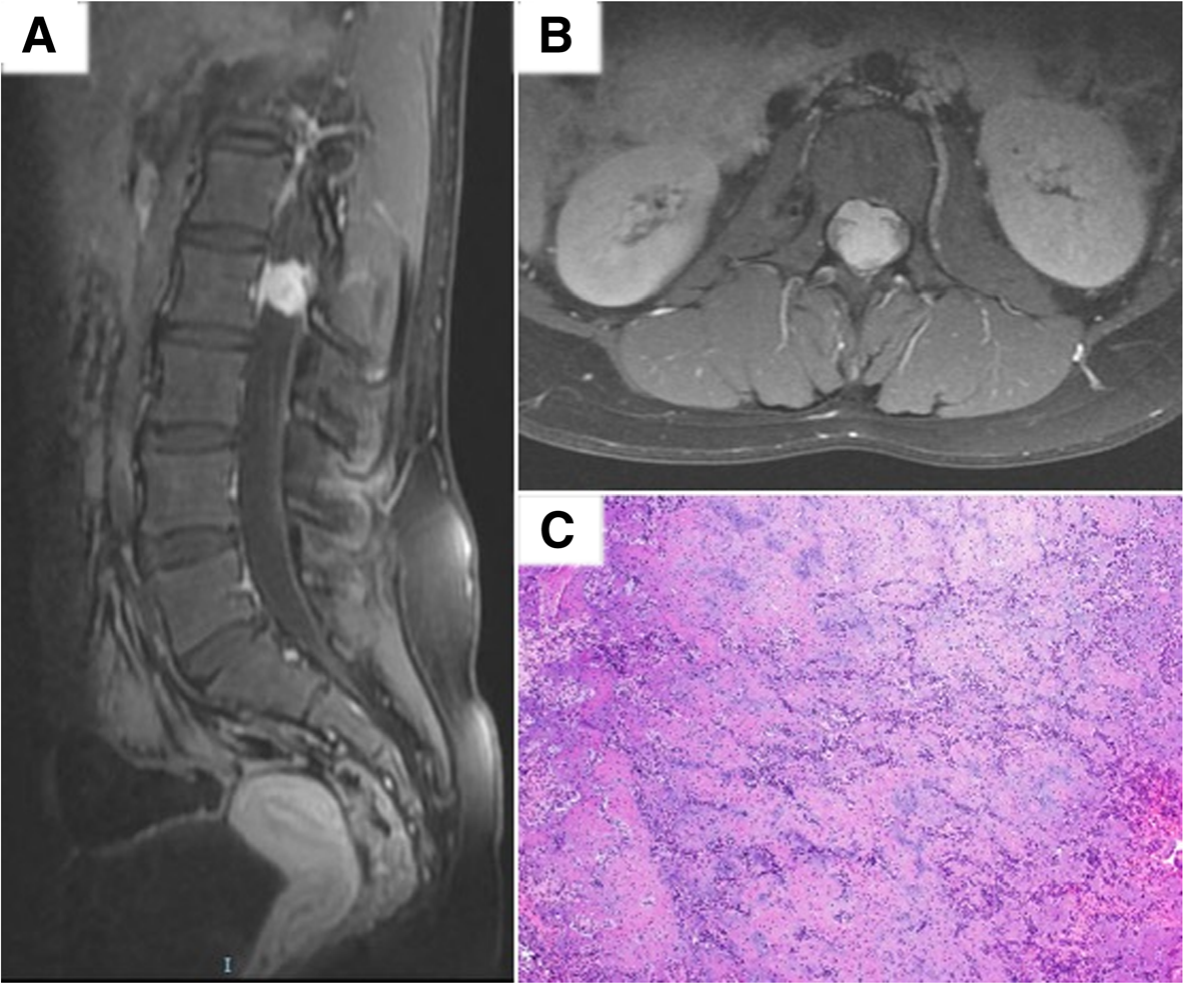
**a** Analysis of anchored multiplex PCR result of the Archer FusionPlex Sarcoma Panel for Patient 1. This illustrates a HEY1 exon 4 and NCOA2 exon 13 fusion, with Reads (#/%) of 86/ 68.3; and a HEY1 exon 4 and NCOA2 exon 14 fusion, with Reads (#/%) of 81/ 30. **b** Analysis of anchored multiplex PCR result of the Archer FusionPlex Sarcoma Panel for Patient 2. This illustrates a HEY1 exon 4 and NCOA2 exon 13 fusion, with Reads (#/%) of 1081/ 92.8; and a HEY1 exon 4 and NCOA2 exon 14 fusion, with Reads (#/%) of 86/ 5.4. The red arrows in both (**a**) and (**b**) indicate the nucleotide location of the gene-specific primers for NCOA2 gene used in the Archer FusionPlex Sarcoma Panel

A previously well 11-year-old female presented with progressively worsening headaches associated with bilateral papilledema. An MRI brain reported a large, heterogeneously enhancing fronto-temporal extra-axial lesion supplied by the right middle meningeal artery. The lesion was resected. Histology showed a mesenchymal chondrosarcoma featuring crowded sheets of primitive spindle to round tumour cells admixed with interspersed islands of neoplastic cartilage demonstrating foci of hyalinization and secondary ossification. Tumour cells were immunoreactive for CD99 but negative for epithelial membrane antigen and progesterone receptor. Ki-67 proliferation index was 1 to 2%. (Fig. 1). The Archer™ FusionPlex Sarcoma Assay detected 2 gene fusion transcripts: HEY1 (exon 4)-NCOA2 (exon 13) and HEY1 (exon 4)-NCOA2 (exon 14) (Fig. 2a and 3).

### Case 2: Lumbar intradural extramedullary mesenchymal chondrosarcoma

**Fig. 4:**
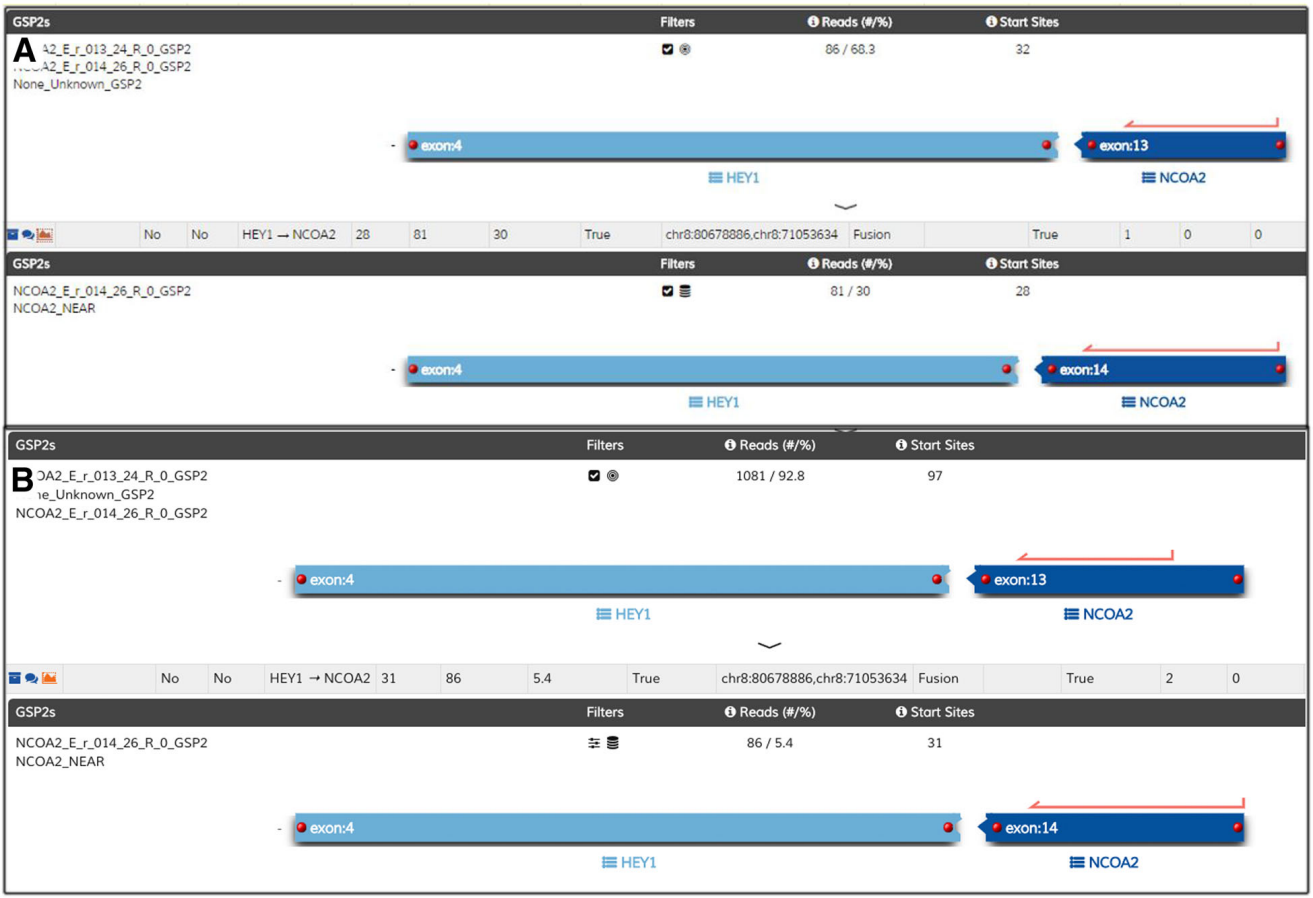
Sanger sequencing of the RT-PCR amplicon products for Patient 1 confirm the presence of both variants (**a**) and (**b**) of HEY1-NCOA2 gene fusion

A 12-year-old female complained of persistent lower back pain associated with bilateral lower limb radicular symptoms over a 4-month duration. The MRI lumbar spine demonstrated an enhancing intradural, extramedullary lesion with adjacent dura thickening at the level of L2. Laminectomy and excision of the lesion was performed. Histology showed a mesenchymal chondrosarcoma featuring round to spindle cells with interspersed cartilage and bone formation. Tumour cells showed diffuse CD99 immunoreactivity and negative staining for epithelial membrane antigen, STAT6 and glial fibrillary acid protein. The Ki-67 index was about 30%. (Fig. 4). Similar to the previous case, the Archer™ FusionPlex Sarcoma Assay detected 2 gene fusion transcripts: HEY1 (exon 4)-NCOA2 (exon 13) and HEY1 (exon 4)-NCOA2 (exon 14) (Fig. 2b).

### Case 3: Thoracic intradural extramedullary Ewing sarcoma

**Fig. 5:**
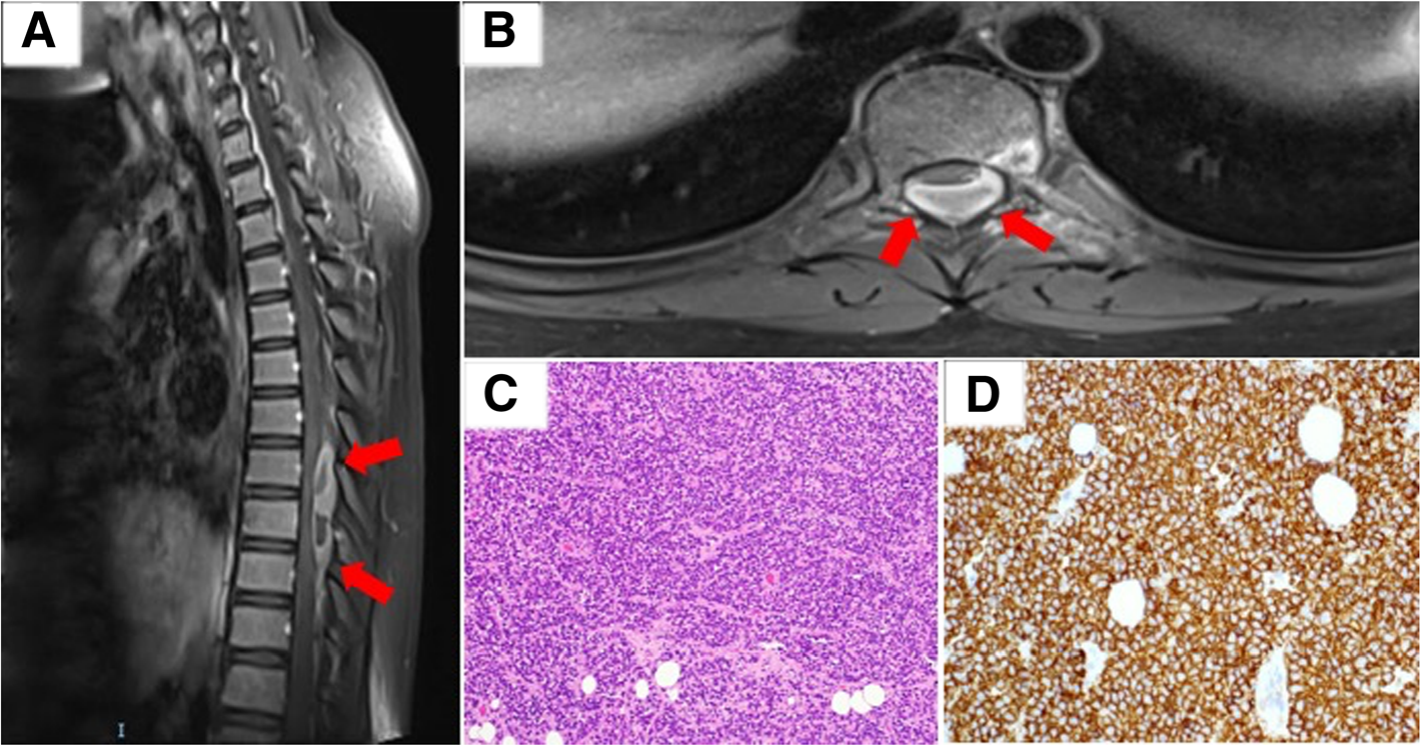
Representative post-contrast T1-weighted MRI images in sagittal (**a**) and axial (**b**) demonstrating a heterogeneously enhancing T6 to T9 extramedullary, extradural soft tissue mass with suggestion of a dural tail. **c** Haematoxylin and eosin stain slide (× 100) illustrating a malignant round cell neoplasm. **d** Immunohistochemistry slide (× 100) shows positivity for CD99

**Fig. 6:**
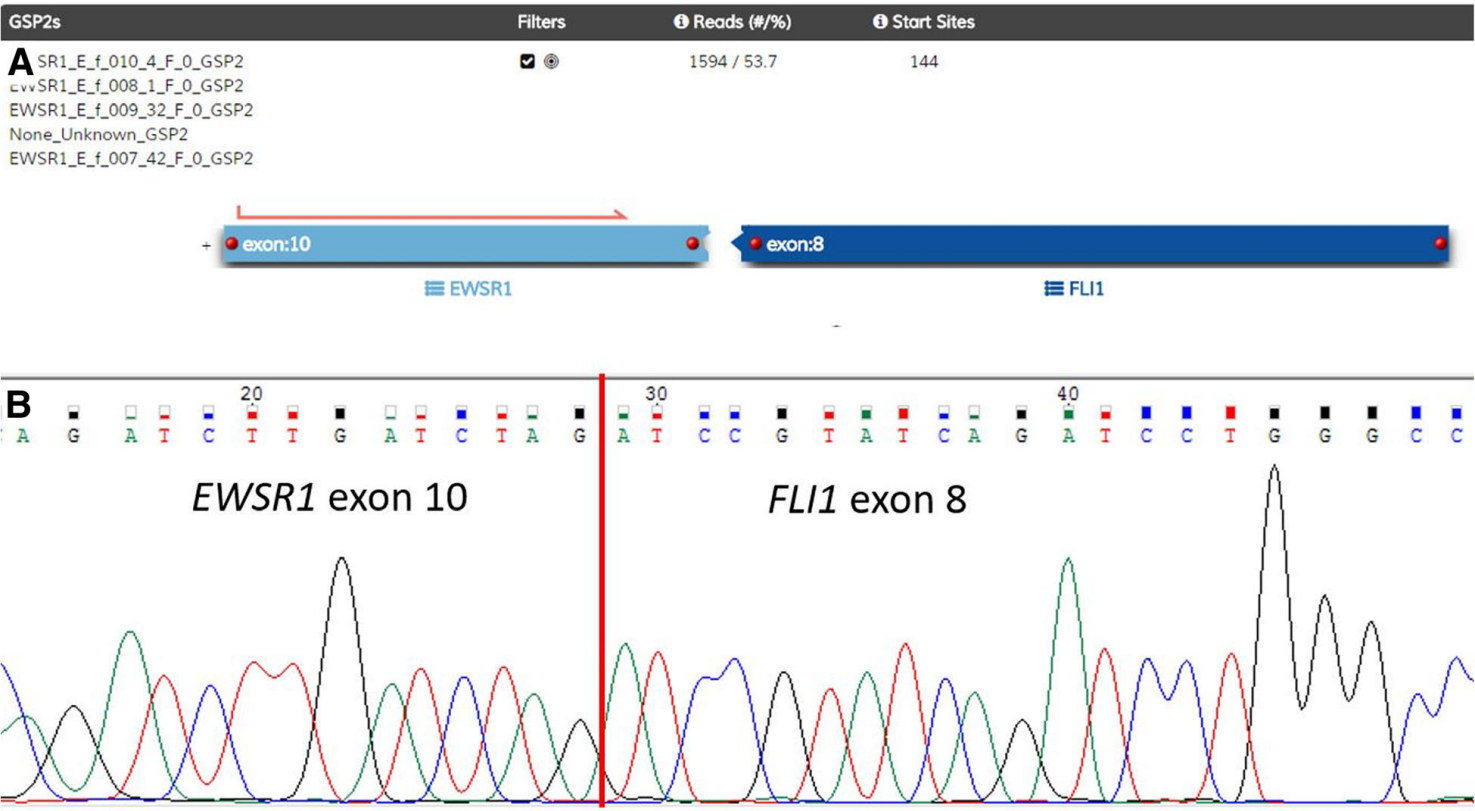
(**a**) Analysis of anchored multiplex PCR result of the Archer™ FusionPlex Sarcoma Panel for Case 3. This shows a EWSR1 exon 10 and FLI1 exon 8 gene fusion with Reads (#/%) of 1594/ 53.7. (**b**) Subsequent Sanger sequencing of the RT-PCR amplicon product confirms the presence of EWSR1 (exon 10)-FLI1(exon 8)

An 11-year-old female presented with a 3-day history of acutely worsening lower limb weakness, numbness, urinary retention and lax anal tone. There was no prior history of associated trauma or injury, and no complaints of back pain. MRI spine revealed an extramedullary extradural soft tissue mass spanning T6 to T9 and causing moderate to severe spinal canal stenosis. This mass was heterogeneously enhancing with suggestion of a dural tail. She underwent emergency T7 to T9 laminectomy and excision of tumour. Histopathology reported a malignant round cell neoplasm with CD99 immunopositivity consistent with Ewing sarcoma (Fig. 5). Fluorescence in situ (FISH) with an EWSR1 break-apart probe was unexpectedly negative. This FISH test was performed twice with different sections of the tumour. However, the Archer™ FusionPlex Sarcoma Assay reported a EWSR1 (exon 10)-FLI1(exon 8) translocation (Fig. 6).

### Study outline and experimental details

This was a single-institution, retrospective study approved by our hospital ethics review board. All patients’ legal guardians (as they are below 21 years old) provided signed informed consent for the research use of their medical data and pathological material obtained as part of their routine clinical data, and publication. The project is exploratory in design, and included 3 patients less than 18 years of age with a histopathological diagnosis of epidural sarcoma.

Total RNA was extracted from macro-dissected tissue sections using the Promega ReliaPrep™ FFPE Total RNA Miniprep System (Promega, USA) as per manufacturer’s protocol. The quantity of extracted RNA was measured using the QuantiFluor® RNA System (Promega, USA). Archer™ FusionPlex Sarcoma Assay is an RNA-based targeted sequencing assay that can identify fusions involving any of 26 sarcoma-related genes (*ALK, CAMTA1, CCNB3, CIC, EPC, EWSR1, FKHR, FUS, GLI1, HMGA2, JAZF1, MEAF6, MKL2, NCOA2, NTRK3, PDGFB, PLAG1, ROS1, SS18, STAT6, TAF15, TCF12, TFE3, TFG, USP6, YWHAE*) without prior knowledge of fusion partners or breakpoints. 150 ng of RNA was used for library preparation utilizing the Archer® FusionPlex® Sarcoma Panel kit (AK00328) following the manufacturer’s protocol (ArcherDX, USA). The prepared library was sequenced using an Ion Torrent PGM™ next-generation sequencer. The Hi-Q™ sequencing kit and Hi-Q™ View CHEF kit were used according to the manufacturer’s protocol (Life Technologies, USA). Data was analyzed by the Archer Data Analysis (version 5.1.0) portal (ArcherDX, USA). Confirmatory RT-PCR and Sanger Sequencing on the amplicon product were performed to confirm the fusion variant. Two separate confirmatory RT-PCR using primers for HEY1/NCOA2 (4–13) – 5′ CGAGATCCTGCAGATGACC 3′ (forward), 5′ GAGGTATCACTGAGTAGGGACTA (reverse)- and HEY1/NCOA2 (4–14) – 5′ CGAGATCCTGCAGATGACC 3′ (forward), 5′ CTGCTGGGTTCCGAATCATA (reverse)- flanking the breakpoint, and Sanger Sequencing on the amplicon products were performed to confirm the fusion variant. For the thoracic epidural tumour, confirmatory RT-PCR using primers – 5′ GAGCGAGGTGGCTTCAATAA 3′ (forward), 5′ GGTTGGCTAGGCGACTG (reverse)- flanking the breakpoint, and Sanger Sequencing on the amplicon product was performed to confirm the fusion variant.

## Discussion

Childhood sarcomas occurring in the CNS children are rare and poorly understood. To begin with, mesenchymal chondrosarcoma (MCS) is an infrequent member of the heterogeneous sarcoma family of tumours [6]. Presently, its developmental mechanisms remain poorly understood. Clinical experience with CNS-related MCS is also extremely limited; nonetheless, local recurrence and distant metastasis has been reported [7]. Presently, CNS-based MCS has only been reflected in surgical case reports and small series in the literature [7–15]. Recently, the HEY1 (exon 4)-NCOA2 (exon 13) gene fusion is reported as a recurrent event unique to all MCS [16, 17]. In this study, we report 2 variant gene fusions HEY1 (exon 4)-NCOA2 (exon 13) and HEY1 (exon 4)-NCOA2 (exon 14) in two of our MCS patients. With reference to current literature, only HEY1 (exon 4)-NCOA2 (exon 13) gene fusion has been previously reported as a recurrent event unique to MCS [16, 17]. However, the concurrent HEY1 (exon 4)-NCOA2 (exon 14) fusion found in both of our CNS cases has not been previously described.

At this point in time, it is hard to postulate if this result is unique to CNS-based MCS patients, or to other MCS tumours found in the rest of the body. In addition, the concurrent occurrence of these 2 transcripts is intriguing. Firstly, there is a possibility that a splicing event has given rise to the two variant gene fusions. Nonetheless, as the breakpoints involve different exons, this is not a usual alternative splicing phenomenon. Next, this phenomenon may be biallelic in that there are two variants of the gene fusion occurring in each of the two copies of the genes. Functional validation at of these findings is required to elucidate their biological meaning. Furthermore, in the context of our patients, there is a role to explore if having 2 aberrant gene fusion variants implies increased oncogenicity in these rare tumours.

Following in the footsteps of scarcity, primary epidural Ewing sarcoma (EWS) is too, very rare in the EWS family of solid tumours. For our third patient, the Archer™ FusionPlex Sarcoma Assay detected EWSR1 (exon 10)-FLI1(exon 8) translocation.. EWS/FLI has at least 10 different isoforms [18]; The most common are: type 1 [EWSR1 (exon 7)-FLI1(exon 6)], type 2 [EWSR1 (exon 7)-FLI1(exon 5)], type 3 [EWSR1 (exon 10)-FLI1(exon 6)] and type 4 [EWSR1 (exon 7)-FLI1(exon 7)] translocations [18]. Here, our result represents an uncommon variant in the cohort of EWSR1-FLI1 translocation breakpoints.

Broadly speaking, non-osseous forms of EWS, particularly those that originate in the epidural space, make up to approximately 20 case reports in the literature [19–21]. Next, EWS is notorious for multiple fusion combinations involving several partner genes [22]. To complicate matters, the specific breakpoint in each partner gene can be variable, resulting in a variety of exon-exon fusion combinations at the transcript level [23]. At this point, we remain uncertain if individual fusion isoforms portend different patient outcomes [24, 25]. However, most are in agreement that detection of translocations at the exon level will have implications for diagnosis, prognosis and treatment of EWS patients [18]. In the context of our patient, the gene fusion EWSR1 (exon 10)-FLI1(exon 8) was investigated using a EWSR1 break-apart probe that could detect a range of EWSR1 gene disruptions. The probes included the translocation region of interest. However, this particular gene fusion was only detectable using NGS methods, and not via FISH. Despite its proven reliability as diagnostic method, FISH is reputed to have a very small risk of false negative results [26]. This technical limitation has been reported to be especially exemplified by EWS [26, 27], as the FISH technique relies on the identification of gene rearrangements to which probes are very specifically directed [18]. For our patient’s case, the consideration may be for multi-color FISH with more than 2 different probes [28]. Under such circumstances, obscure translocations that lead EWSR1 insertion into partner genes may be more readily detectable. However, this particular type of FISH testing is not readily available at our institution. Hence, this case highlights the utility of novel genomic advancements as a clinical tool in challenging cases. It should too, be emphasized that selected patients may proceed to be studied by more than one diagnostic modality, especially if the initial test is negative and the clinical features point to a high probability of genetic aberrations [29].

In general, paediatric cancers appear to be the consequence of chromosomal rearrangements, rather than mutation events [3]. Detection and characterization of gene fusions has been of great importance for clinical purposes, as well as for understanding tumorigenesis [30]. Furthermore, it has been reported that sarcoma patients whose tumours were interrogated by NGS as part of their clinical management received the option of access to targeted agents in their treatment [31]. This is because a difference in clinical outcome may be predicted by one or more of the following: firstly, incidence of a specific fusion; next, the presence of one or more variants of a fusion; or finally, the presence of one of a heterogeneous group of unrelated fusions [32, 33]. More significantly, as the cases in discussion are rare paediatric tumours, an isolated fusion found in a single patient can be important, especially for future therapeutic targeting [3]. Identification of specific fusion transcripts, including unusual variants of gene-related splices may help in the development of novel therapeutics to block their aberrant activity in cancer cells [34].

### Study critique and future directions

The authors acknowledge that there are limitations that should be highlighted in this study. First and foremost, this is a retrospective study with a small number of cases. However, it should be emphasized that primary paediatric epidural sarcomas are extremely rare. Hence, given the infrequency of such patients, every case should be regarded as significant. Moving forward, the role of functional studies to determine the clinico-pathological relevance of gene fusions in this subset of location-specific sarcomas is paramount.

## Conclusion

In summary, the authors describe the molecular experience of 3 paediatric epidural sarcomas, in corroboration with current disease understanding and techniques. The identification of gene fusions may offer insights into the biology of these exceptionally rare neoplasms; and more importantly, bear clinical relevance for future targeted therapy. The authors advocate multi-disciplinary, collaborative efforts at a global level to benefit children afflicted with these malignant tumors.

## Abbreviations

CNS: Central nervous system
EWS: Ewing sarcoma
MCS: Mesenchymal chondrosarcoma
MRI: Magnetic resonance imaging
NGS: Next-generation sequencing
